# A Longitudinal Study Simultaneously Exploring the Carriage of APEC Virulence Associated Genes and the Molecular Epidemiology of Faecal and Systemic *E. coli* in Commercial Broiler Chickens

**DOI:** 10.1371/journal.pone.0067749

**Published:** 2013-06-25

**Authors:** Kirsty Kemmett, Tom Humphrey, Steven Rushton, Andrew Close, Paul Wigley, Nicola J. Williams

**Affiliations:** 1 Institute of Infection and Global Health, Department of Infection Biology, School of Veterinary Science, The University of Liverpool, Neston, Cheshire, United Kingdom; 2 Institute of Infection and Global Health, Department of Epidemiology and Population Health, School of Veterinary Science, The University of Liverpool, Neston, Cheshire, United Kingdom; 3 School of Biology, Newcastle University, Newcastle Upon Tyne, United Kingdom; Iowa State University, United States of America

## Abstract

Colibacillosis is an economically important syndromic disease of poultry caused by extra-intestinal avian pathogenic *Escherichia coli* (APEC) but the pathotype remains poorly defined. Combinations of virulence-associated genes (VAGs) have aided APEC identification. The intestinal microbiota is a potential APEC reservoir. Broiler chickens are selectively bred for fast, uniform growth. Here we simultaneously investigate intestinal *E. coli* VAG carriage in apparently healthy birds and characterise systemic *E. coli* from diseased broiler chickens from the same flocks. Four flocks were sampled longitudinally from chick placement until slaughter. Phylogrouping, macro-restriction pulsed-field gel electrophoresis (PFGE) and multi-locus sequence typing (MLST) were performed on an isolate subset from one flock to investigate the population structure of faecal and systemic *E. coli*. Early in production, VAG carriage among chick intestinal *E. coli* populations was diverse (average Simpson's D value  = 0.73); 24.05% of intestinal *E. coli* (n = 160) from 1 day old chicks were carrying ≥5 VAGs. Generalised Linear models demonstrated VAG prevalence in potential APEC populations declined with age; 1% of *E. coli* carrying ≥5 VAGs at slaughter and demonstrated high strain diversity. A variety of VAG profiles and high strain diversity were observed among systemic *E. coli*. Thirty three new MLST sequence types were identified among 50 isolates and a new sequence type representing 22.2% (ST-2999) of the systemic population was found, differing from the pre-defined pathogenic ST-117 at a single locus. For the first time, this study takes a longitudinal approach to unravelling the APEC paradigm. Our findings, supported by other studies, highlight the difficulty in defining the APEC pathotype. Here we report a high genetic diversity among systemic *E. coli* between and within diseased broilers, harbouring diverse VAG profiles rather than single and/or highly related pathogenic clones suggesting host susceptibility in broilers plays an important role in APEC pathogenesis.

## Introduction

Colibacillosis is a syndromic disease of birds characterised by fibrinous lesions around visceral organs caused by a group of extraintestinal pathogenic *Escherichia coli* (ExPEC) known as avian pathogenic *E. coli* (APEC). Airsacculitis, cellulitis, pericarditis, perihepatitis and respiratory distress are among the most commonly associated signs of colibacillosis in broiler (meat producing) chickens [1]. Extraintestinal *E. coli* infections are a considerable economic burden on the global poultry industry due to increased mortality rates during rearing and rejection of carcasses at slaughter. Despite a number of studies aimed at elucidating the APEC pathotype, it remains poorly defined. Genes involved in bacterial adhesion, invasion, toxin production, serum survival and iron acquisition have all been shown to contribute to APEC pathogenesis [2], [3], [4], [5], [6], [7]. It is likely that combinations of virulence associated genes (termed VAG profiles or virulotypes) are needed to give rise to pathogenic *E. coli*, as no single gene has been exclusively associated with APEC. A recent study demonstrates APEC strains arise from multiple *E. coli* lineages following the acquisition of distinct VAGs, highlighting the potential high genetic diversity among these bacteria [8]. Serotyping has been used as a method for identifying APEC but several authors suggest it fails to discriminate APEC and avian faecal *E. coli* and a significant proportion of *E. coli* is untypable [9].

Previous studies have identified the gastrointestinal microbiota as a potential reservoir for APEC infection [5], [9]. It has been shown that infection follows either inhalation of contaminated faecal dust followed by septicaemia or via active gut translocation [10], [11]. Intestinal *E. coli* carrying numerous VAGs maybe referred to as ‘potential’ APEC (pAPEC) populations and their presence is likely to pose an increased risk to systemic disease.

Commercial broiler chickens are selectively bred for their efficient and uniform growth. Despite its' commercial importance, relatively little work has exclusively focused on colibacillosis in broiler chickens [5], [6], [9], [12]. The gastrointestinal tract of a young animal is a rich ecological niche ideal for bacterial colonisation and subsequent microbial succession. The outcomes of host-microbial interactions are influenced by host (age, immunity), the microbe (microbiota, VAGs) and environmental factors [13], [14]. Initially, gut colonisation of production birds can be influenced by: vertical transmission, the hatchery environment, handling, transportation [15], [16], [17]. Once on farm, birds are exposed to a different rearing environment, dietary changes and vaccinations (see materials and methods).

It is currently unknown how APEC VAG carriage among intestinal *E. coli* populations change with respect to bird age. Thus, the current study aims to address several new questions: to determine whether there are significant changes with time in the intestinal pAPEC reservoir among avian faecal *E. coli* populations; if certain *E. coli* strains, VAGs and/or profiles are selected for in the gastrointestinal tract through time and how this relates to the strains and VAG profiles seen among systemic *E. coli* isolated from birds, which die as a consequence of APEC infection.

## Materials and Methods

### Ethics statement

The following protocol involved the (non invasive) collection of faecal samples (using sterile cotton swabs) following excretion and no approval under the Animals (Scientific Procedures) Act (1986) was needed. No birds were culled for the purpose of this study and all dead birds intended for post-mortem examination were collected on the first daily welfare walk conducted by farmers. The study was approved by the University of Liverpool Committee on Research Ethics: Physical Interventions sub-committee (reference RETH000448) with the mandatory condition that any serious adverse events be reported to the sub-committee within 24 hours. The study was conducted in strict accordance with the University of Liverpool Research Governance policies and permission for sampling on the broiler farms was granted by the farms.

### 2.1 Longitudinal sample collection

Two consecutive flock cycles on two standard commercial broiler chicken farms in the UK were visited once to twice weekly. The sampling described below commenced from the day the chicks were placed in rearing houses and was completed approximately 3 days before the first de-population event (∼32–35 days). Approximately 30% of the flock is removed at depopulation to allow farmers to conform to end-of-life stocking density standards. The flocks used in this study were routinely vaccinated as industrial practice in the UK: Avian Pneumovirus (7 days old), Infectious Bronchitis virus (14 days old) and Infectious Bursal Disease (16 days old). The flocks did not receive any veterinary treatment. All *E. coli* isolates collected during the course of this study are available upon request.

#### 2.1.1 Gut E. coli population VAG carriage

At each visit, 20 fresh faecal swabs were collected at random from different areas of the broiler house floor. Each swab was cultured onto eosin-methylene blue agar (EMBA) and incubated overnight at 37°C. From each plate, eight randomly selected colonies typical of *E. coli* were subcultured onto nutrient agar to obtain pure cultures and incubated overnight at 37°C. All media used were obtained from LabM (IDG) Ltd (Bury, UK). *E. coli* identification was confirmed using a PCR targeting the *uidA* gene [18]. One colony, representing each of the eight isolates, was pooled in 600 µl of 20% (w/v) Chelex-100 in 10 mM Tris-HCl, 1 mM EDTA, pH 8.0 (Bio-Rad, Hertfordshire, UK). DNA was extracted from each pooled sample using a modified protocol described previously [19]. Briefly, 600 µl of Chelex 100 containing pooled colonies was incubated at 95°C for 10 min. Samples were centrifuged at 10,000 rpm for 2 min and 50 µl of supernatant was added to 250 µl of sterile double distilled water.

As a means of screening faecal *E. coli*, largely expected to be non-pathogenic, each pooled DNA extract was screened for four VAGs previously associated with avian *E.coli* pathogenesis; *iss, tsh, iucC* and *cvi*, using a multiplex PCR [2]. Primers were obtained from Eurofins MWG operon (Germany); all PCR constituents used in this study were supplied by Thermo Scientific, Surrey. All four primer sequences are given in Skyberg *et al* [2]. Briefly, each 50 µl reaction contained: 12 µl of 25 mM MgCl_2_, 21.3 µl sterile water, 5 µl 10×PCR buffer, 4 µl of 20 mM dNTPs, 0.3 µl of each 100 pmol forward and reverse primer, 0.3 µl 5 U/µl Taq polymerase and 5 µl template DNA. Thermocycler conditions were: initial denaturation 95°C for 5 min; nine cycles of 95°C for 60 sec, 55°C for 30 sec, 72°C for 60 sec; twenty eight cycles of 94°C for 30 sec, 55°C for 30 sec, 72°C for 30 sec with a final extension 72°C for 7 min. The mixture was held at 4°C. PCR products were subject to electrophoresis on a 2% agarose gel in tris–acetate buffer (TAE) at 150 volts for 60 min alongside a Superladder-Low 100 bp ladder (Thermo Scientific, Surrey). When a sample pool was positive for ≥3 of the 4 genes, a new Chelex-100 preparation was made for each individual isolate within the pool as described above. Pooled samples with fewer than 3 VAGs were discarded. The individual isolate DNA templates were then screened for 10 VAGs; *astA, iss, irp2, iucD, papC, tsh, vat, cvi, sitA* and *ibeA*. Three separate PCR assays were performed; one multiplex PCR previously described by Ewers *et al* [3] and two single PCR assays for *ibeA* and *sitA* outlined by Timothy *et al* [20]. Briefly, for a 25 µl multiplex PCR, 4 µl of 25 mM MgCl_2_, 13.9 µl sterile water, 2.5 µl 10×PCR buffer, 0.5 µl 20 mM dNTPs, 0.1 µl of each 100 pmol forward and reverse primers, 0.5 µl 5 U/µl Taq polymerase and 2 µl DNA template were used. Multiplex PCR thermocycler conditions were as follows: initial denaturation 94°C for 3 mins followed by 25 cycles of: 94°C for 30 secs, 58°C for 30 secs, 68°C for 3 mins with a final extension 72°C for 10 mins. The mixture was held at 4°C. Each individual PCR contained 1 µl DNA template, 1 µl of each primer (100 pmol) and 22 µl of 1.1×Reddymix PCR mastermix with 1.5 mM MgCl_2_. Thermocycler conditions for *sitA* and *ibeA* were identical; 95°C for 12 min and 25 cycles of: 94°C for 30 sec, 63°C for 30 sec, 68°C for 3 min; 72°C for 10 min with a final hold 4°C. PCR products were subject to electrophoresis as above. The presence or absence of the 10 VAGs produced a series of 10 numbers, which denoted the VAG profile for each isolate (presence ‘1’ or absence ‘0’). Isolates carrying ≥5 VAGs were classified as pAPEC.

#### 2.1.2 Post-mortem examination of dead broiler chickens

As well as faecal sample collection throughout rearing, from week 2 onwards, at each faecal sampling time point, 8 dead birds were collected from the first welfare walk of the day for post-mortem examination. To minimise the detection of systemic *E. coli* resulting from a loss of intestinal integrity following death, only birds identified as recently dead were included. Birds were only selected for post-mortem examination if they did not show signs of extensive pecking, had not been trodden on (flattened appearance) and/or did not have broken legs or other obvious injury. For all birds, any observed classic colibacillosis characteristics were recorded including; ascites, airsacculitis, cellulitis, enlarged spleen, pericarditis and perihepatitis [1]. For each bird, up to 1 gram of the following tissues were collected; heart, kidney, liver, lung and spleen using sterile forceps and scalpels. An equal volume of sterile phosphate buffered saline (PBS) was added to each sample and tissues were homogenised using a Biomaster Micro-stomacher 80 (Steward, UK) for 60 seconds at high speed. 50 µl of the homogenate was streaked onto EMBA and incubated overnight at 37°C. Eight *E.coli* colonies were picked, re-plated onto nutrient agar and incubated overnight at 37°C. All isolates were immediately subjected to a full screen of all 10 virulence genes using the assays described above and each isolate was given a corresponding VAG profile.

##### *2.1.2.2 Statistical analysis*

Collected data were analysed using multiple statistical tests. Intestinal *E. coli* VAG profile diversity at each sampling time point was calculated using Simpson's diversity index (D). Generalised linear models (GLMs) were used to investigate the relationship between VAG profile diversity and time. Several different statistical measures were used a) the Pearson's correlation coefficient from VAG profile diversity data and the detection of potential APEC isolates b) the P-value obtained from the Fisher's exact test to assess the distribution of VAG genes between faecal and systemic *E. coli* population. Associations were considered statistically significant if the calculated P-value was <0.05.

### 2.2 Phylogenetic typing

Faecal and systemic isolates collected from one of the four flock cycles underwent further molecular analysis by phylogenetic typing. Two hundred and sixteen faecal and 35 systemic *E. coli* were analysed.

Isolates were assigned to 1 of 4 *E. coli* phylogenetic groups (A, B1, B2 or D) using a triplex PCR targeting *chuA, yjaA* and the DNA fragment TSPE4.C2 [21]. Each 25 µl PCR reaction contained: 1 µl of template DNA extract, 1 µl of each forward and reverse 100 pmol primer (Eurofins MWG operon, Germany) and 22 µl of 1.1×Reddymix with 1.5 mM MgCl_2_. Thermocycler conditions were as follows: initial denaturation at 94°C for 4 mins; 30 cycles of; 5 secs at 94°C and 10 secs at 59°C with final extension at 72°C for 5 mins. The reaction mixture was held at 4°C. PCR products were subject to electrophoresis as stated above. Phylogenetic group classification was based on the combination of *chuA*, *yjaA* and TSPE4.C2: A (*chuA* ^−^, TSPE4.C2^−^, *yjaA* ^+^), B1 (*chuA^−^*, TSPE4.C2^+^, *yjaA^−^*), B2 (*chuA* ^+^, TSPE4.C2^−/+^, *yjaA* ^+^) and D (*chuA* ^+^, TSPE4.C2^−/+^, *yjaA^−^*).

### 2.3 Macro-restriction pulsed-field gel electrophoresis

Two hundred and twenty two faecal and 48 systemic *E. coli* were analysed using PFGE. The PFGE protocol used was based on the standardised Pulsenet Rapid *E. coli* method [22] with slight modifications. During sample preparation, plugs were incubated for 2 h at 54°C with vigorous shaking at 175 rpm and for sample digestion each sample was incubated for 2 h with 50U of *Xba*1 restriction enzyme (Roche products Ltd, Hertfordshire) at 37°C. Samples were run on a 1% 0.5X Tris-Borate running buffer (TBE) (Life technologies, UK) agarose universal (alpha laboratories, Hampshire) with 0.5X TBE running buffer for 20 hours at 14°C, at 6 V/cm^2^ with the initial switch time of 2.2 s and final switch time of 54.2 s in a CHEF-DRIII PFGE system. A Lambda ladder PFGE marker (New England Biolabs, Ipswich, MA, USA) was run on each gel. The gel was stained in an ethidium bromide solution (500 µl ethidium bromide in 500 ml 0.5X TBE running buffer) for 25 mins and visualised under UV using a transilluminator. Samples which failed PFGE analysis, were re-tested with a longer proteinase K incubation period; 24 h at 54°C with vigorous shaking at 175 rpm. Image analysis was performed using BioNumerics version 4.0 and Dendrograms were constructed using the Unweighted Pair Group Method with Arithmetic Mean (UPGMA).

### 2.4 Multi-Locus sequence typing (MLST)

Fifty *E. coli* isolates from intestinal and systemic sites were analysed by MLST. Genomic template DNA was prepared using the Chelex-100 DNA extraction method as previously described [19]. Seven house-keeping genes were targeted for PCR; adenylate kinase (*adk*), fumerate hydratase (*fumC*), DNA gyrase (*gyrA*), isocitrate dehydrogenase (*icd*), malate dehydrogenase (*mdh*), adenylosuccinate dehydrogenase (*purA*) and the ATP/GTP binding motif (*recA*) [23]. All primer sequences and a detailed protocol are given by Wirth *et al* [23]. For this present study, the PCR based protocol was modified slightly and each 25 µl reaction contained: 0.5 µl of each forward and reverse primer (20 pmol), 23 µl of 1.1×Reddymix with 1.5 mM MgCl_2_ and 1 µl of template DNA (Chelex 100 extractions). The PCR conditions included an initial denaturation at 95°C for 2 mins, 30 cycles of; 95°C for 1 mins, target specific primer annealing temperature for 1 mins (outlined in Wirth *et al*, 2006) and a final extension at 72°C for 5 mins. PCR success was confirmed by running products on a 1.5% agarose gel in TAE buffer for 30 mins at 150 v. The remaining product was cleaned using a 20% (w/v) polyethylene glycol (PEG_8000_), 2.5 M NaCl (Yorkshire Bioscience Ltd, UK) precipitation protocol. Cleaned PCR products were sequenced commercially (Macrogen, Korea) with 1∶15 diluted sequencing primers (same as amplification primers). Sequences were analysed using ChromasPro version 1.5 (Technelysium, Australia) and MEGA 5.05 [24] and submitted to the Achtman *E. coli* MLST online database (http://mlst.ucc.ie/mlst/dbs/Ecoli). To determine the genetic relatedness of our STs and those previously submitted to the online database, Burst (version 3) diagrams were constructed following the online instructions (http://eburst.mlst.net/).

## Results

### 3.1 E. coli carriage of virulence-associated genes in healthy broiler chickens

A total of 420 *E. coli* pools was obtained from apparently healthy birds from two crops on two farms, between May and July 2011 and following initial screening, 119 were positive for ≥3 of the 4 targeted VAGs. Thus a total of 952 isolates were assigned a VAG profile out of 3360. Generally, fewer pooled samples met the threshold as birds aged.

**Figure 1:**
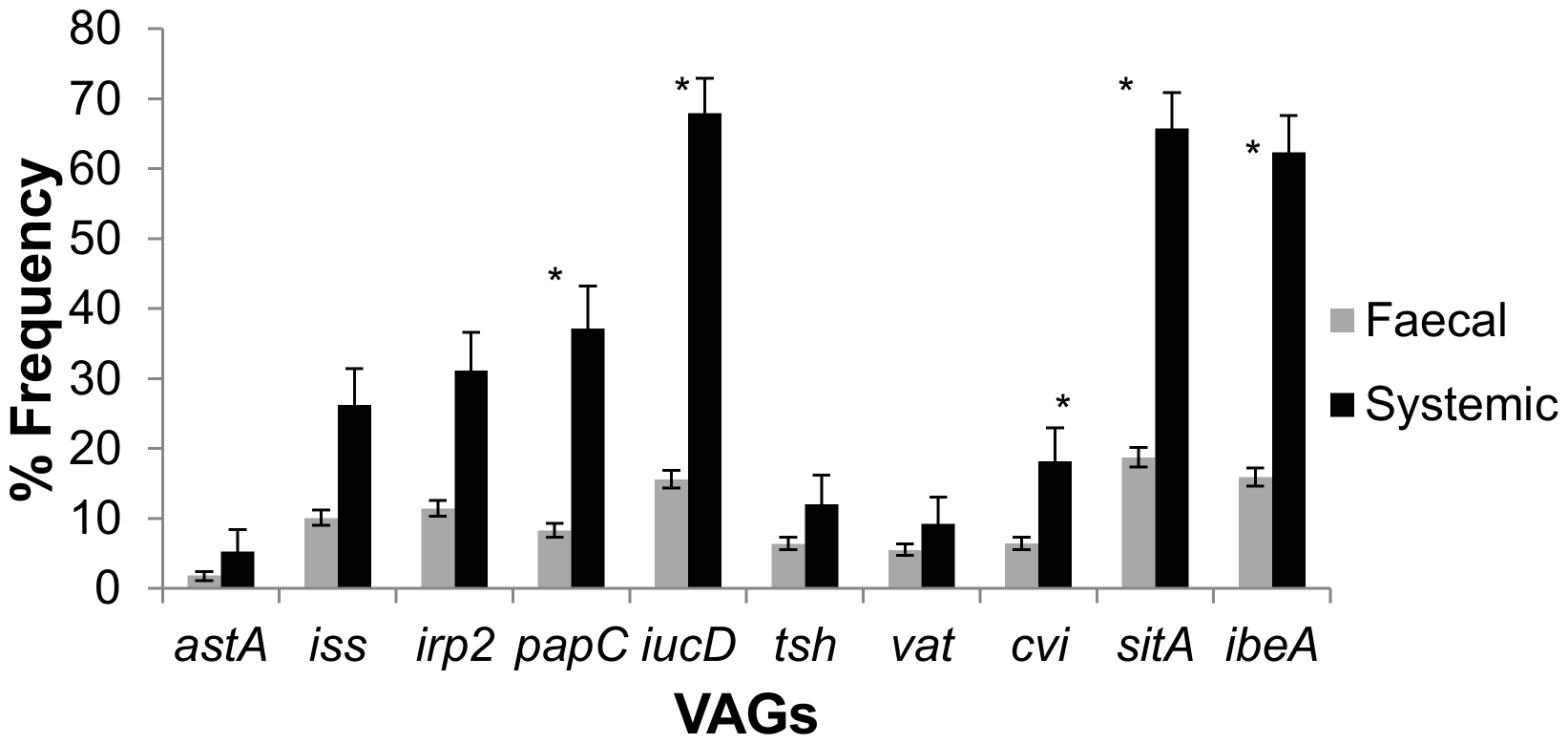
Comparison of faecal and systemic *E.* *coli* VAG carriage. Upper and lower bound 95% confidence intervals indicate statistically significant differences between VAG carriages by the two populations. Fisher's exact test indicates that *irp2, papC, iucD, cvi, sitA* and *ibeA* are significantly more associated with systemic *E. coli* (p<0.05) denoted in figure by *.

Overall, VAGs were more frequently associated with systemic *E. coli* populations than faecal ones (Figure 1). For individual intestinal *E. coli* isolates, the *sitA* gene was the most commonly detected VAG, ranging between 0.68% and 20.57% prevalence, with an average detection of 8.51% over each sampling point for all flocks. Toxin encoding genes (*astA* and *vat*) were the least frequently detected in these populations; 0.00–11.25% (average 1.12%) and 0.00–9.38% (average 2.11%) for *astA* and *vat* respectively over the four flock cycles. Genes associated with iron acquisition, *sitA*, *iucD* and *irp2*, were commonly carried by individual isolates averaging 5.10% and 7.34% for *irp2* and *iucD* respectively.

**Figure 2:**
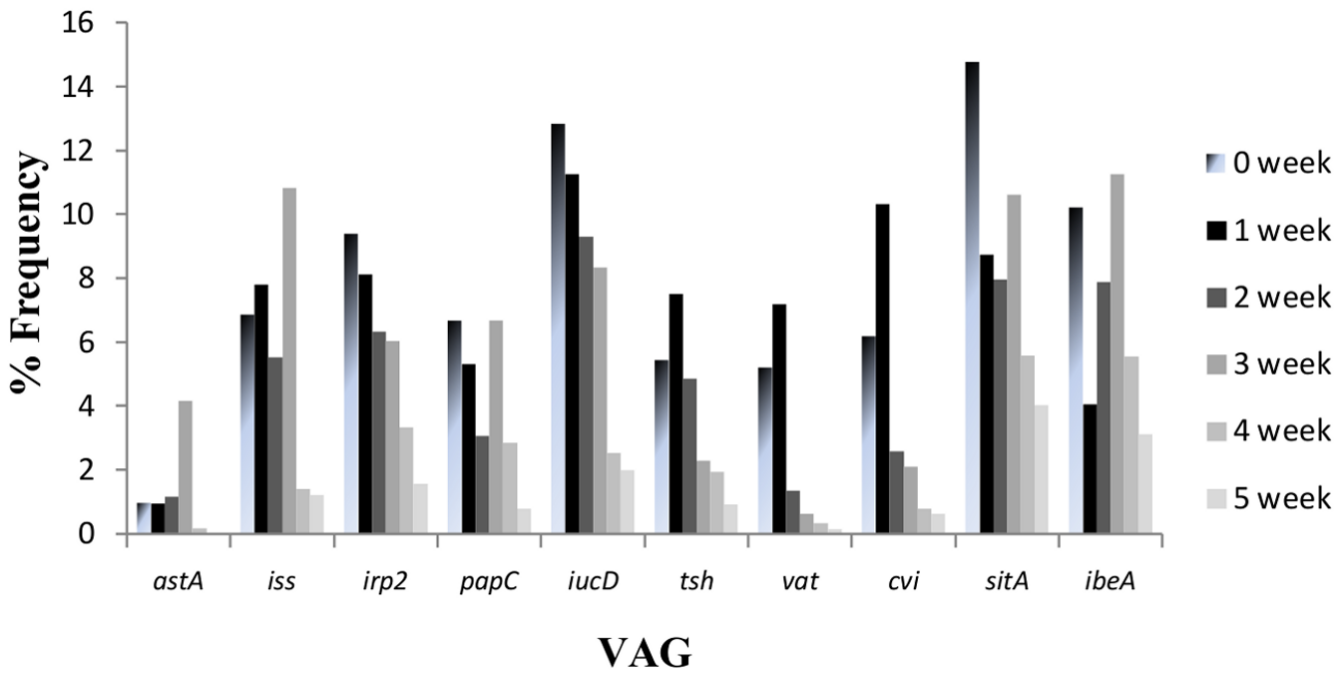
Average percentage frequency of VAGs. (inclusive of all flocks). Average percentage frequencies of 10 VAGs were calculated and plotted against time, from t = 0 (arrival) to t  =  week 5 (depopulation). Overall, VAGs appear to decline with time, with a peak in detection at week 3 for *iss*, *sitA* and *ibeA*. Iron acquisition genes *irp2* and *iucD* consistently decline with time.

The frequency at which the invasion-related gene, *ibeA*, was detected varied, ranging from 0.6% to 17.73% over the 4 flock cycles. At t = 0, *ibeA* detection ranged from 5–14.10%. Over the first week, the level of *ibeA* detection decreased, before peaking between weeks 2–3 (approximately 13%) and then declining once again (to 3.12%) towards week 5. A similar trend was also observed for *iss* detection (Figure 2).

**Figure 3:**
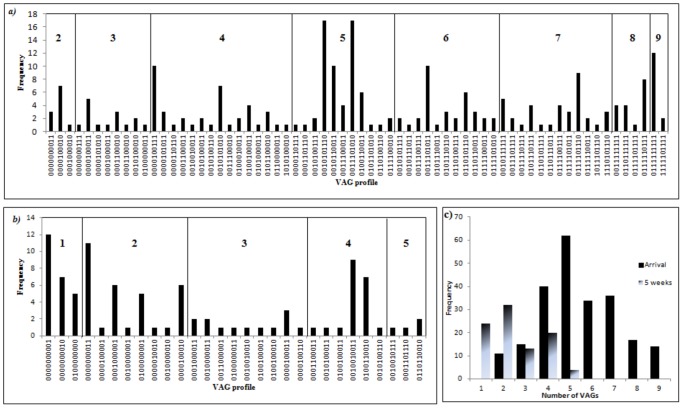
VAG profile diversity for all flocks. *a*) Shows the VAG profiles identified at t = 0 (arrival of chicks). Profiles consisting of 4 VAGs were the most diverse, with differences in iron acquisition genes being the most abundant, while profiles 0010101110 (*irp2* ^+^, *papC^+^*, *va*t^+^,*cvi* ^+^, *sitA* ^+^) and 0011101010 (*irp2* ^+^, *iucD* ^+^, *papC* ^+^, *va*t^+^, *sitA* ^+^) both with 5 VAGs were the most common profile identified *b*) Shows the VAG profiles identified at t  =  week 5. VAG profile diversity had declined over time. Most diversity was detected with the possession of 3 VAGs. No isolates carried more than 5 VAGs *c*) Comparison of total number of VAGs carried by individually tested *E. coli* at t = 0 and 5. Profile 206 (0000000000) excluded from both graphs. Not all profiles were represented in all four cycles.

VAG profiles (P-) were created based on observed combinations of the 10 different VAGs targeted (a systematic numbering system). A total of 206 different unique profiles were observed in the faeces of apparently healthy broiler chickens; P-1 (*astA, iss, irp2, iucD, papC, tsh, cvi, vat, sitA, ibeA*: 0000000001) represents the carriage of i*beA* only, whereas P-206 is assigned to isolates carrying none of the targeted genes. P-206 was the most common profile detected in all flocks and its level of detection increased with time perhaps suggesting a positive selection for non-pathogenic traits within an intestinal population. Figure 3 shows the frequencies of detection for different profiles at t = 0 and t = week 5.

#### 3.1.1 Changes in VAG profile diversity with respect to farm/flock and time

When the VAG data were analysed for individual farms and flocks; F1C1 (farm 1; cycle1), F2C1, F1C2 and F2C2, a total of 57, 45, 86 and 112 different VAG profiles were identified, respectively. Sixty two out of 206 different profiles (30.10%) were detected on >1 farm/flock, while 69.9% were only identified on one farm. Despite farm/flock individual VAG profile frequency differences, a common trend was observed with respect to time.

**Figure 4:**
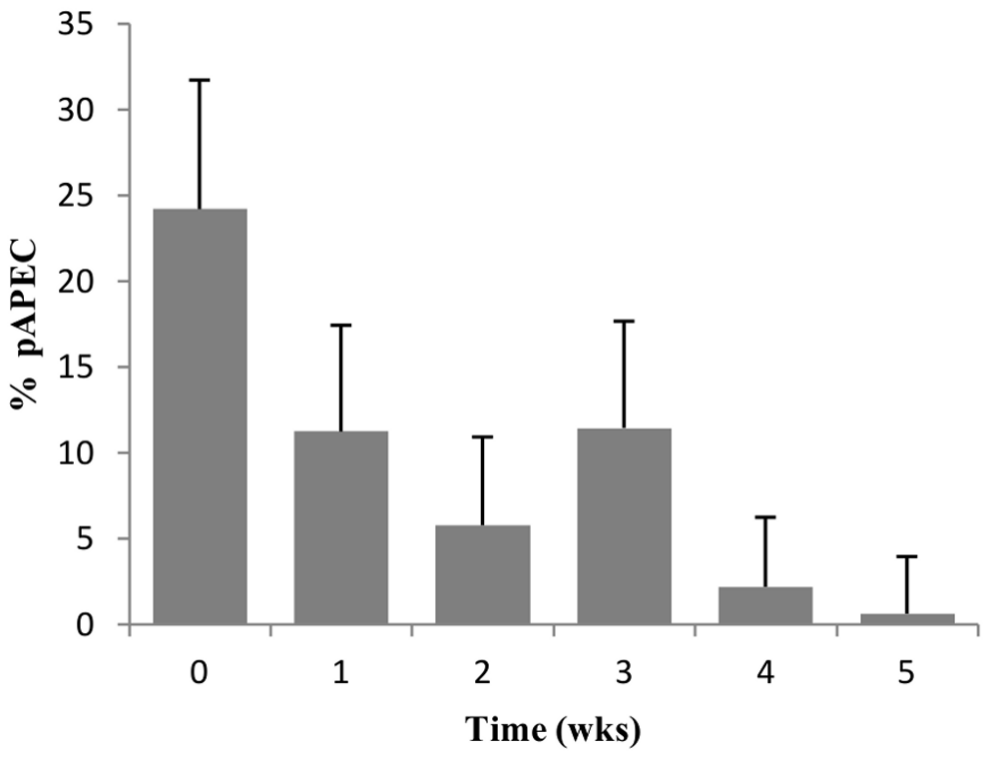
Average percentage of pAPEC with respect to time. At weekly intervals the average percentage of potential APEC, defined by the carriage of ≥5 VAGs, from the total faecal *E.coli* population was calculated. At each time point, 160 faecal *E. coli* were assessed. 95% upper confidence interval error bars shown. Overall, there is a general decline with time; the average detection frequency at placement of chicks (week 0) was 24.05% and only 1% by week 5.

On average, 24.05% of *E. coli* isolates screened from the gastrointestinal tract of chicks at t = 0 (placement) carried at least 5 of the 10 VAGs (termed pAPEC) (Figure 4). The *sitA* gene was consistently the most frequently detected VAG from all four flock cycles on the two farms.

Simpson's diversity index (D) was used to compare the profile diversity at each week of production for the second flock cycles on both farms; D values are shown in Table 1. Generalised linear models confirmed the significant effect of time on VAG profile diversity (p<0.05). Overall, VAG profile diversity declined through time, with a common peak at week 3 of production.

**Table 1:** Simpson's diversity index for VAG profile diversity through time.

	D value	
Week	F1	F2
**0**	0.683	0.779
**1**	0.683	0.359
**2**	0.438	0.582
**3**	0.704	0.686
**4**	0.070	0.307
**5**	0.391	0.200

Simpson's diversity index (D) was used to compare VAG profile diversity through time in the second flock cycles of farm 1 (F1) and farm 2 (F2). Overall, profile diversity decreases with time, with a peak at week 3.

As birds aged, the percentage frequency of pAPEC in the gastrointestinal tract declined. Prior to the first depopulation event at 5 weeks of production, only 1% of *E. coli* carried ≥5 VAGs.

An average decrease of 12.97% in pAPEC from t = 0 to week 1 was detected, followed by a further 5.47% decrease between weeks 1 and 2 (Figure 4).

### 3.2 Longitudinal analysis of systemic E. coli carriage of virulence-associated genes

On average, over the four flocks, 39.1% of dead birds (n = 128) collected on the first daily welfare walk showed signs of colibacillosis and systemic *E. coli* was identified. Three hundred and twenty four isolates were virulotyped. Figure 4 shows the distribution of VAG frequencies between both faecal and systemic *E. coli* populations. Fisher's exact test was used to assess the frequency differences between the faecal and systemic populations; *irp2, papC, iucD, cvi, sitA* and *ibeA* genes were significantly associated with systemic *E. coli* populations (p<0.05); *astA*, *vat*, *iss* and *tsh* were not (p>0.05).

Sixty three different profiles were detected among the systemic *E. coli* collected. Thirteen of the 63 profiles (20.63%) were found on more than one farm. Fifty eight of 324 isolates (17.90%) carried no VAGs (P-206). P-15 (*ibeA* ^+^, *iucD^+^*, *sitA^+^*) was the second most frequent profile (9.88%). However, this was only identified on F1C1. Of the profiles which were found on more than one farm, 46.26% accounted for profiles with ≥4 VAGs; in all these profiles at least 50% of the genes detected were involved in iron acquisition. None of the tested isolates carried more than 7 VAGs. Observed VAG profile diversity was not correlated with the number of *E. coli* investigated (p>0.05), suggesting sample size variation has not influenced profile detection and thus the reported level of diversity. Over the four flock cycles, 36.4–80% of VAG profiles identified in systemic isolates were also identified at least once in faecal isolates collected before and/or at the same time from apparently healthy birds during the same cycle. Nineteen profiles out of 63 were unique amongst systemic isolates. However, only one of these profiles was identified on more than one occasion (P-221; *iss* ^+^, *irp2* ^+^, *papC* ^+^, *iucD* ^+^). Overall, there were no profiles, which appeared to be wholly significantly associated with diseased birds; the large VAG profile diversity seen amongst systemic isolates suggests that specific VAG profile alone are not responsible for disease in commercial broiler chickens.

### 3.3 Phylogenetic analysis

Table 2 shows the assignment of 216 faecal *E. coli* and 35 systemic *E. coli* collected from F1C2 to the four phylogenetic groups and 1 subgroup. If no amplification occurred for any of the 3 targets, isolates were assigned to subgroup A_0_ [25], [26].

**Table 2:** Assignment of faecal and systemic *E. coli* to phylogenetic groups.

	Number of isolates (% frequency)				
Source	A	A0	B1	B2	D
**Faecal**	85 (39.35)	21 (9.72)	1 (0.46)	5 (2.31)	104 (48.15)
**Systemic**	11 (31.43)	14 (40.00)	0 (0.00)	1 (2.86)	9 (25.71)
**Total**	96	35	1	6	113

216 faecal *E. coli* and 35 systemic *E. coli* from vital organs were typed using the Clermont *et al* triplex PCR and assigned to 1 of 5 phylogenetic groups. Those isolates which showed no amplification of any of the 3 targets, yet confirmed to be *E. coli* were assigned to subgroup A0. Group D was the most frequently detected phylogenetic group among faecal population, while A0 (untypable) was the most common group among systemic isolates. B2 and D have been previously associated with more pathogenic *E. coli*, however they only accounted for 28.57% of systemic isolates in this study.

With the exception of week 2 (22.50%), group D was the most frequently detected phylogenetic group among faecal isolates. In week 2, Group A was most frequently detected phylogenetic group (58.75%). The screening-based protocol of faecal isolates would have led to sampling bias towards ones containing VAGs and therefore possibly group B2 and D isolates. There are no obvious changes in phylogenetic groups through time.

Fourteen of 35 (40%) systemic isolates grouped into phylogenetic group A_0_. Previously pathogenic associated phylogenetic groups D and B_2_ represented 25.71% and 2.86% of systemic isolates respectively. Results suggest that no distinct phylogenetic group accounts for systemic *E. coli*.

### 3.4 Macro-restriction PFGE analysis

Two hundred and twenty two faecal and 48 systemic *E. coli* isolated from the same flock were analysed by PFGE to look for changes in gut population through time, common strain types associated with systemic *E. coli* and to relate genetic background to the carriage of VAGs. One hundred and sixty six faecal and 35 extraintestinal isolates were successfully digested and dendrograms constructed.

A dendrogram constructed from the pulsotypes of 48 *E. coli* isolated from faeces at t = 0 shows large strain diversity and no apparent association between strain type and VAG carriage. The 48 isolates fell into 5 groups with 80% similarity. There appears to be no retained strain type correlated with time.

**Figure 5:**
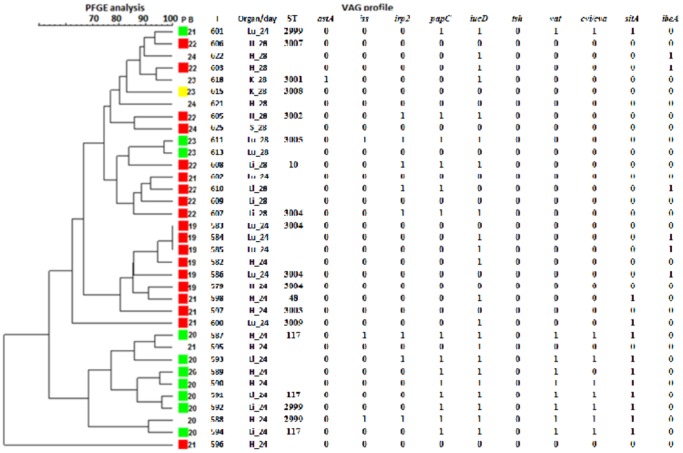
Dendrogram constructed using DICE for systemic *E.* *coli*. (tolerance 5%) (minimum height >0.0%, minimum surface >0.0%)(0.0–100% coefficient) for XbaI PFGE. A dendrogram showing the strain diversity amongst systemic E. *coli* harbouring APEC VAGs constructed using BioNumerics software by unweighted pair group method with Arithmetic mean. The dendrogram also shows; phylogenetic group (P) (green  =  D; red  =  A; yellow  =  B2; blue  =  B1), isolate (I), organ and age of bird at isolation (H  =  heart: K  =  kidney: Li  =  liver: Lu  =  lung; S  =  spleen), MLST sequence type (ST) and VAG profiles. The dendrogram shows the clustering of ST 117 and 2999 isolates (excluding 601) which by PFGE analysis are ∼60% different from other isolates. Several ST 3004 were identified and these potentially show the acquisition of 2 Iron acquisition genes (*irp2* and *iucD*) while other ST 3004 isolates have no VAGs (isolates 579 and 583).

Thirty-five systemic *E. coli* belonged to 10 groups with 80% similarity (Figure 5), suggesting diverse strain diversity amongst systemic isolates. The dendrogram also highlights the isolation of multiple strain types from individual diseased birds and the presence of similar strain types amongst faecal and systemic isolates.

### 3.5 MLST analysis

To assess underlying clonal association between isolates with VAGs in faecal and diseased bird populations, 24 faecal *E. coli* (8 with ≥5 VAGs, 8 with <5 VAGs and 8 with 0 VAGs) and 23 extraintestinal *E. coli* (11 with ≥5 VAGs, 7 with <5 VAGs and 5 with 0 VAGs) were submitted to the MLST online database (http://mlst.ucc.ie/mlst/dbs/Ecoli). All results are shown in table 3.

**Table 3:** Observed faecal and systemic *E. coli* MLST Sequence types categorised by VAG carriage.

VAGs	Site of isolation	
	Faeces	Systemic
**0**	2990, 2991, 2992, 2993, 2994, 2995, 2996, 2997	3003, 3004 (2), 3007, 3008
**<5**	2980, 2981, 2982, 2983, 2987, 2988, 2989	3001, 3002, 3004 (2), 3005, 3006, 3009, 10
**≥5**	352 (3), 2978, 2984, 2985, 2986, 3010	117 (3), 2998, 2999 (4), 3000

(n) * = * ST observation frequency. All faecal *E. coli* belonged to newly identified sequence types (ST) excluding ST-352. Interestingly, all ST-352 isolates harboured more than 5 VAGs with the following profiles:1) *astA* ^+^, *irp2* ^+^, *papC* ^+^, *iucD* ^+^, *vat* ^+^, *cvi* ^+^, *sitA* ^+^ 2) *iss* ^+^, *irp2* ^+^, *papC* ^+^, *iucD* ^+^, *vat* ^+^, *cvi* ^+^, *sitA* ^+^ 3) *iss* ^+^, *irp2* ^+^, *papC* ^+^, *iucD* ^+^, *vat* ^+^, *cvi* ^+^, *sitA* ^+^ and they did not group with other *E. coli* in the online database. Systemic *E. coli* analysis identified 3 ST–117 and 4 ST- 2999 isolates; however ST-2998 and ST-3000 did not cluster with the other two STs in this category.

In total, 33 new sequence types were identified, 6 of which were single locus variants (SLV) of ST-10. This was the only clonal complex (CC-10) in which faecal and systemic isolates were shown to be related.

Interestingly, 3 of the 24 faecal isolates were identified as ST-352 and all carried more than 5 VAGs 1) *astA* ^+^, *irp2* ^+^, *papC* ^+^, *iucD* ^+^, *vat* ^+^, *cvi* ^+^, *sitA* ^+^ 2) *iss* ^+^, *irp2* ^+^, *papC* ^+^, *iucD* ^+^, *vat* ^+^, *cvi* ^+^, *sitA* ^+^ 3) *iss* ^+^, *irp2* ^+^, *papC* ^+^, *iucD* ^+^, *vat* ^+^, *cvi* ^+^, *sitA* ^+^. ST-352 did not cluster in any of the other faecal or systemic isolates in the constructed burst diagrams.

Four ST-2999 isolates (representing 22.22% of systemic isolates tested) were isolated from two diseased birds, ST-2999 is a SLV of the emerging pathogenic clone ST-117 [27]. All four ST-2999 isolates carried ≥5 VAGs and no ST-2999 isolates were identified among the faecal population. Furthermore, the genetic relatedness of ST-2999 and ST-117 is highlighted in their general clustering in constructed PFGE dendrograms (Figure 5). ST-48 (CC-10) and ST-10 (CC-10) were also amongst those already known STs identified in systemic populations. ST-3004 was identified only among systemic isolates. ST-3004 isolates were found to differ in the number of VAGs they carried; no VAGs (isolate 579 and 583), 1 (isolate 586) and 3 (isolate 607). Two out of the 3 VAGs are involved in iron acquisition (*irp2* and *iucD*) (Figure 5).

## Discussion

To our knowledge, this is the first study to address the longitudinal diversity of intestinal *E.coli* populations with a focus on APEC VAG carriage, while simultaneously characterising systemic *E. coli* isolated from visceral organs of diseased birds in UK broiler flocks.

Previous work suggests the clonal nature of *E. coli* makes it possible to associate certain lineages with ExPEC status which could help elucidate a “typical” APEC [28], [29], [30], [31], [32]. The *E. coli* genome has a high degree of plasticity whilst retaining a level of clonality resulting from recombination events of short mobile elements in genome ‘’hotspots’’, often these elements contain VAGs [12], [33], [34], [35]. A similar observation was made recently regarding the clonality of extended β-lactamase producing *E. coli* [36]. Research suggests APEC arise from the acquisition of VAGs and certain lineages may be more accepting of incoming genetic elements and thus pathogenic [37], [38], [39]. In the current study, MLST identified a new sequence type (ST-2999) among the systemic isolates carrying ≥5 VAGs. ST-2999 is an SLV of ST-117, a potentially emerging pathogenic ST previously associated with retail chicken and human disease [27], [40]. ST-117 was also identified among the systemic isolates. PFGE allows for more refined comparisons between isolates and here confirmed the genetic relatedness between these isolates compared to the other systemic ones. However, the overall high level of strain diversity among systemic *E. coli* isolated from diseased birds; the lack of correlation with VAG carriage and the identification of multiple strains as opposed to a single clone in one bird perhaps suggests the opportunistic nature of certain *E. coli* [41]. Additionally, this perhaps suggests differences in broiler susceptibility compared to other avian species (layer hens and turkeys) where single clones of APEC have been identified [8], [12], [29]. As only a subset of isolates were subjected to genetic analysis, it is possible that we have underestimated the level of diversity present. It is clear a high level of diversity exists.

The intestinal *E. coli* population of birds has previously been identified as an APEC reservoir [5], [9]. The findings from this study further support this with 36.4–80.0% of systemic VAG profiles also being identified among faecal *E. coli* of the same flock.

*E. coli* is one of the first bacterial species to colonise the neonatal gut before succession [42], [43], [44]. A large proportion of pAPEC contributed to early colonisation of the neonatal chick (24.05% of tested population). Sources of such *E. coli* include: parent flock (vertical transmission), hatchery environment, human handling and transportation equipment [15], [16], [17]. Yassin *et al* (2009) correlated first week mortalities with hatchery and breeder age, highlighting the potential important influence of these factors [45]. Interestingly, despite all four flocks in this study being sourced from different hatcheries, the level of observed pAPEC at this stage was comparable. Past studies have shown that the possession of VAGs could be advantageous in microbial gut populations offering commensalism fitness advantages [46], [47], [48]. The positioning of VAGs on mobile genetic elements would allow for their selective maintenance within populations [7], [12], [34].

As birds aged, both VAG profile diversity and the detection of pAPEC declined; by the last week of production, 1% of the population sampled were classified as pAPEC. Furthermore, as birds exceeded 3 weeks of age there was a noticeable decline in the proportion of pooled samples reaching the 3 VAG threshold outlined in our sampling protocol, suggesting a negative selection in the avian gut. Younger birds have been shown to possess a more diverse microbiota compared to that of older birds, likely to be due to rapid initial opportunistic colonisation of an available ecological environment; with age microbial succession and interspecies convergence occur [14], [49]. The bottle necking of VAG diversity and pAPEC with microbial succession may represent the persistence of stronger colonisers and the loss of more transient strains. One hypothesis is that different VAGs offer selective advantages at different stages of development [34]. A note of caution is required, as our list of VAGs is not an exhaustive list of APEC-associated virulence genes.

Irrespective of time, *sitA* was the most frequently detected VAG in this study. The *sitABCD* encoded transporter regulates iron and manganese transport and provides increased resistance to oxidative stress [50]. This mechanism could be advantageous among competing gastrointestinal populations and during inflammation [14]. Additionally, the redundancy of iron acquisition systems is thought to be advantageous in environmental survival [51]. Interestingly our study identified multiple ST-3004 isolates which differed in their possession of VAGs namely ones involved in iron acquisition (*irp2* and *iucD*). Could this be the result of gene transfer and acquisition?

The *ibeA* gene was frequently detected among intestinal *E. coli* populations of young birds. The *ibeA* gene encodes a 50 kDa protein thought to aid brain microvascular epithelial adherence and invasion [4]. The exact mechanism of Ibe proteins remains to be determined but it has been shown to modulate type 1 fimbriae [52]. The advantage of possessing *ibeA* while in the gut remains unknown; it could relate to the increased survival of attached *E. coli*, particularly in a transient inflammatory environment [4], [53].

The 10 VAGs selected for this study do not represent an exhausted list of APEC determinants [54]. For future work, an investigation published after this study was carried out presents a new virulotyping protocol offering vastly improved error margins in APEC detection, ideal for epidemiological studies[55]. Based on the literature the APEC pathotype is likely to contain a mix of iron acquisition genes and those encoded on plasmids [6], [9]. This was reflected in our chosen panel of VAGs. It was necessary to add a level of bias to the faecal sampling given the ubiquitous nature of *E. coli* in the gastrointestinal tract allowing practical detection of the proportion of the population that are potentially pathogenic. Such sampling is technically demanding and labour intensive. The 4 VAGs used in the initial screening were selected based on their high prevalence among APEC strains; *iss* (∼83%), *iucC* (75%), *tsh* (53–63%) and *cvi* (63%) [56], [57]. This panel allowed for the detection of as many potential APEC as possible given the limitations in screening the large number of samples. All calculations regarding the ‘proportion of potentially pathogenic *E. coli’* was calculated using the entire population sampled, i.e. the original number of *E. coli* picked before initial PCR screening.

The avian host also contributes to shaping the microbiota. Lu *et al* (2003) described a more stable microbiota between 2 and 4 weeks of age in fast growing birds, reflecting the current study which observed more consistent levels of pAPEC between weeks 4 and 5 [14]. Immunological changes during host development are likely to contribute to changes in the microbiota; heterophil function (avian polymorphonuclear neutrophils (PMNs)) has been shown to be lacking in day old chicks [58]. Crhanova *et al* (2011) reported transient gut physiological inflammation in 4 day old chicks, while the cellular immune responses to *Salmonella* Typhimurium of 1 day old and 1 week old chicks have been shown to be markedly different, suggesting rapid immunological changes in early life [14], [49], [59], [60]. It is likely that a combination of host (immunity and vaccination), microbial (microbiota composition, VAG carriage) and environmental (feed, production systems) changes has contributed to the changes in pAPEC observed in this study, highlighting the importance of host-microbial interactions [61], although this needs to be looked at more closely,. It would be of interest to determine causes of death in the first 48–72 hours of life; a period of limited heterophil function, often the point of highest mortality during commercial rearing and as noticed in this study the point in production where APEC VAGs are at the greatest prevalence in the avian gut [45].Exploratory analysis to elucidate the contribution of environmental factors to the observed changes in pAPEC is ongoing.

In summary, we have shown colonisation of the broiler gut by pAPEC to occur before chicks are placed and as broilers age the populations shift while appearing to bottle neck in VAG carriage diversity. The reasons for this remain to be determined. Our work supports that of others, identifying the avian gut as an APEC reservoir, but did not find a predominant APEC pathotype in the flocks studied. The identification of highly diverse systemic *E. coli* populations rather than single or highly related clones perhaps suggests the broiler chicken and its susceptibility is a major contributor to disease manifestation. Further work is required (i.e. molecular analysis on more isolates, elucidation of contributing impacting factors to pAPEC dynamics), but this study offers the first insight into the temporal movement and dynamics of *E. coli* in the avian host and offers a new approach to deciphering APEC.

To conclude, the concept of an APEC pathotype is arguably fundamentally flawed in broilers.

## References

[1] Barnes HJ, Gross WB (1999) Colibacillosis. In: W.B.Gross, editor. Diseases of Poultry. 10th ed: Iowa state university press, Ames.pp. 131–141.

[2] Skyberg JA, Horne SM, Giddings CW, Wooley RE, Gibbs PS, et al (2003) Characterizing avian Escherichia coli isolates with multiplex polymerase chain reaction. Avian Diseases 47: 1441–1447. 14708994 10.1637/7030

[3] Ewers C, Janssen T, Kiessling S, Philipp HC, Wieler LH (2005) Rapid detection of virulence-associated genes in avian pathogenic Escherichia coli by multiplex polymerase chain reaction. Avian Diseases 49: 269–273. 16094833 10.1637/7293-102604R

[4] Germon P, Chen YH, He L, Blanco JE, Bree A, et al (2005) ibeA, a virulence factor of avian pathogenic Escherichia coli. Microbiology-Sgm 151: 1179–1186. 10.1099/mic.0.27809-0 15817785

[5] McPeake SJM, Smyth JA, Ball HJ (2005) Characterisation of avian pathogenic Escherichia coli (APEC) associated with colisepticaemia compared to faecal isolates from healthy birds. Veterinary Microbiology 110: 245–253. 16150559 10.1016/j.vetmic.2005.08.001

[6] Rodriguez-Siek KE, Giddings CW, Doetkott C, Johnson TJ, Nolan LK (2005) Characterizing the APEC pathotype. Veterinary Research 36: 241–256. 15720976 10.1051/vetres:2004057

[7] Kariyawasam S, Johnson TJ, DebRoy C, Nolan LK (2006) Occurrence of pathogenicity island IAPEC-01 genes among Escherichia coli implicated in avian colibacillosis. Avian Diseases 50: 405–410. 17039841 10.1637/7462-102705R.1

[8] Francis Dziva HH, Connor TR, van Diemen PM, Prescott G, Langridge GC, et al.. (Published online ahead of print (December 2012)) Sequencing and functional annotation of 1 avian pathogenic Escherichia coli serogroup O78 strains reveals the evolution of E. coli lineages pathogenic for poultry via distinct mechanisms. Infection and Immunity. 10.1128/IAI.00585-12 PMC3584874 23275093

[9] Ewers C, Antao EM, Diehl I, Philipp HC, Wieler LH (2009) Intestine and Environment of the Chicken as Reservoirs for Extraintestinal Pathogenic Escherichia coli Strains with Zoonotic Potential. Applied and Environmental Microbiology 75: 184–192. 18997030 10.1128/AEM.01324-08 PMC2612213

[10] Dominick MA, Jensen AE (1984) Colonization and persistence of Escherichia-coli in axenic and monxenic turkeys American Journal of Veterinary Research. 45: 2331–2335. 6240953

[11] Nagaraja KV, Emery DA, Jordan KA, Sivanandan V, Newman JA, et al (1984) Effect of ammonia on the quantitative clearance of Escherichia-coli from lungs, air sacs, and livers of turkeys aerosol vaccinated against Escherichia-coli. American Journal of Veterinary Research 45: 392–395. 6370056

[12] Johnson TJ, Siek KE, Johnson SJ, Nolan LK (2006) DNA sequence of a ColV plasmid and prevalence of selected plasmid-encoded virulence genes among avian Escherichia coli strains. Journal of Bacteriology 188: 745–758. 16385064 10.1128/JB.188.2.745-758.2006 PMC1347294

[13] Morris JG, Potter M (1997) Emergence of new pathogens as a function of changes in host susceptibility. Emerging Infectious Diseases 3: 435–441. 9368786 10.3201/eid0304.970404 PMC2640070

[14] Lu JR, Idris U, Harmon B, Hofacre C, Maurer JJ, et al (2003) Diversity and succession of the intestinal bacterial community of the maturing broiler chicken. Applied and Environmental Microbiology 69: 6816–6824. 14602645 10.1128/AEM.69.11.6816-6824.2003 PMC262306

[15] Christensen JP, Chadfield MS, Bojesen AM, Bisgaard M (2003) Investigations on the clonality of E. coli infections in industrial poultry production. Proceedings from the XIII Congress of the World Veterinary Poultry Association,. Denver, USA. 83.

[16] Petersen A, Christensen JP, Kuhnert P, Bisgaard M, Olsen JE (2006) Vertical transmission of a fluoroquinolone-resistant Escherichia coli within an integrated broiler operation. Veterinary Microbiology 116: 120–128. 16650662 10.1016/j.vetmic.2006.03.015

[17] Fasenko GM, Christopher EEOD, McMullen LM (2009) Spraying hatching eggs with electrolyzed oxidizing water reduces eggshell microbial load without compromising broiler production parameters. Poultry Science 88: 1121–1127. 10.3382/ps.2008-00359 19359703

[18] McDaniels AE, Rice EW, Reyes AL, Johnson CH, Haugland RA, et al (1996) Confirmational identification of Escherichia coli, a comparison of genotypic and phenotypic assays for glutamate decarboxylase and beta-D-glucuronidase. Applied and Environmental Microbiology 62: 3350–3354. 8795225 10.1128/aem.62.9.3350-3354.1996 PMC168131

[19] Walsh PS, Metzger DA, Higuchi R (1991) Chelex-100 as a medium for simple extraction of DNA for PCR-based typing from forensic material. Biotechniques 10: 506–513. 1867860

[20] Timothy S, Shafi K, Leatherbarrow AH, Jordan FTW, Wigley P (2008) Molecular epidemiology of a reproductive tract-associated colibacillosis outbreak in a layer breeder flock associated with atypical avian pathogenic Escherichia coli. Avian Pathology 37: 375–378. 18622852 10.1080/03079450802216579

[21] Clermont O, Bonacorsi S, Bingen E (2000) Rapid and simple determination of the Escherichia coli phylogenetic group. Applied and Environmental Microbiology 66: 4555–4558. 11010916 10.1128/aem.66.10.4555-4558.2000 PMC92342

[22] Ribot EM, Fair MA, Gautom R, Cameron DN, Hunter SB, et al (2006) Standardization of pulsed-field gel electrophoresis protocols for the subtyping of Escherichia coli O157: H7 Salmonella, and Shigella for PulseNet. Foodborne Pathogens and Disease 3: 59–67. 16602980 10.1089/fpd.2006.3.59

[23] Wirth T, Falush D, Lan RT, Colles F, Mensa P, et al (2006) Sex and virulence in Escherichia coli: an evolutionary perspective. Molecular Microbiology 60: 1136–1151. 16689791 10.1111/j.1365-2958.2006.05172.x PMC1557465

[24] Tamura K, Peterson D, Peterson N, Stecher G, Nei M, et al (2011) MEGA5: Molecular Evolutionary Genetics Analysis Using Maximum Likelihood, Evolutionary Distance, and Maximum Parsimony Methods. Molecular Biology and Evolution 28: 2731–2739. 21546353 10.1093/molbev/msr121 PMC3203626

[25] Escobar-Paramo P, Grenet K, Le Menac'h A, Rode L, Salgado E, et al (2004) Large-scale population structure of human commensal Escherichia coli isolates. Applied and Environmental Microbiology 70: 5698–5700. 15345464 10.1128/AEM.70.9.5698-5700.2004 PMC520916

[26] Escobar-Paramo P, Clermont O, Blanc-Potard AB, Bui H, Le Bouguenec C, et al (2004) A specific genetic background is required for acquisition and expression of virulence factors in Escherichia coli. Molecular Biology and Evolution 21: 1085–1094. 15014151 10.1093/molbev/msh118

[27] Mora A, Lopez C, Herrera A, Viso S, Mamani R, et al (2012) Emerging avian pathogenic Escherichia coli strains belonging to clonal groups O111:H4-D-ST2085 and O111:H4-D-ST117 with high virulence-gene content and zoonotic potential. Veterinary Microbiology 156: 347–352. 22112854 10.1016/j.vetmic.2011.10.033

[28] Tivendale KA, Logue CM, Kariyawasam S, Jordan D, Hussein A, et al (2010) Avian-Pathogenic Escherichia coli Strains Are Similar to Neonatal Meningitis E. coli Strains and Are Able To Cause Meningitis in the Rat Model of Human Disease. Infection and Immunity 78: 3412–3419. 20515929 10.1128/IAI.00347-10 PMC2916289

[29] Johnson TJ, Kariyawasam S, Wannemuehler Y, Mangiamele P, Johnson SJ, et al (2007) The genome sequence of avian pathogenic Escherichia coli strain O1: K1: H7 shares strong similarities with human extraintestinal pathogenic E-coli genomes. Journal of Bacteriology 189: 3228–3236. 17293413 10.1128/JB.01726-06 PMC1855855

[30] Manges AR, Johnson JR (in press) Food-borne origins of Escherichia coli causing extraintestinal infections. Clinical infectious diseases. 10.1093/cid/cis502 22615330

[31] Ewers C, Janssen T, Kiessling S, Philipp HC, Wieler LH (2004) Molecular epidemiology of avian pathogenic Escherichia coli (APEC) isolated from colisepticemia in poultry. Veterinary Microbiology 104: 91–101. 15530743 10.1016/j.vetmic.2004.09.008

[32] Ozawa M, Baba K, Asai T (2010) Molecular typing of avian pathogenic Escherichia coli O78 strains in Japan by using multilocus sequence typing and pulsed-field gel electrophoresis. J Vet Med Sci 72: 1517–1520. 20625238 10.1292/jvms.10-0174

[33] Ochman H, Lawrence JG, Groisman EA (2000) Lateral gene transfer and the nature of bacterial innovation. Nature 405: 299–304. 10830951 10.1038/35012500

[34] Tenaillon O, Skurnik D, Picard B, Denamur E (2010) The population genetics of commensal Escherichia coli. Nature Reviews Microbiology 8: 207–217. 20157339 10.1038/nrmicro2298

[35] Touchon M, Hoede C, Tenaillon O, Barbe V, Baeriswyl S, et al.. (2009) Organised Genome Dynamics in the Escherichia coli Species Results in Highly Diverse Adaptive Paths. Plos Genetics 5. 10.1371/journal.pgen.1000344 PMC2617782 19165319

[36] Ewers C, Bethe A, Semmler T, Guenther S, Wieler LH (2012) Extended-spectrum ss-lactamase-producing and AmpC-producing Escherichia coli from livestock and companion animals, and their putative impact on public health: a global perspective. Clinical Microbiology and Infection 18: 646–655. 22519858 10.1111/j.1469-0691.2012.03850.x

[37] Mellata M, Touchman JW, Curtiss R III (2009) Full Sequence and Comparative Analysis of the Plasmid pAPEC-1 of Avian Pathogenic E-coli chi 7122 (O78:K80:H9). Plos One 4. 10.1371/journal.pone.0004232 PMC2626276 19156210

[38] Smith JL, Fratamico PM, Gunther NW (2007) Extraintestinal pathogenic Escherichia coli. Foodborne Pathogens and Disease 4: 134–163. 17600482 10.1089/fpd.2007.0087

[39] Brown PK, Curtiss R (1996) Unique chromosomal regions associated with virulence of an avian pathogenic Escherichia coli strain. Proceedings of the National Academy of Sciences of the United States of America 93: 11149–11154. 8855324 10.1073/pnas.93.20.11149 PMC38299

[40] Vincent C, Boerlin P, Daignault D, Dozois CM, Dutil L, et al (2010) Food Reservoir for Escherichia coli Causing Urinary Tract Infections. Emerging Infectious Diseases 16: 88–95. 20031048 10.3201/eid1601.091118 PMC2874376

[41] Dziva F (2010) Deciphering the infection biology of avian pathogenic Escherichia coli: role of experimental infection models. In: Vilas AM-, editor; 2010. pp. 746–753.

[42] Syed SA, Abrams GD, Freter R (1970) Efficiency of various intestinal bacteria in assuming normal functions of enteric flora after association with germ-free mice. Infection and Immunity 2: 376–386. 16557849 10.1128/iai.2.4.376-386.1970 PMC416020

[43] Barnes EM, Barnum DA, Harry EG, Mead GC (1972) Intestinal flora of chicken in period 2 to 6 weeks of age, with particular reference to anaerobic bacteria. British Poultry Science 13: 311-&. 10.1080/00071667208415953 4555258

[44] Salanitr JP, Fairchil IG, Zgornick YD (1974) Isolation, culture characteristics, and identification of anaerobic bacteria from chicken cecum. Applied Microbiology 27: 678–687. 4596749 10.1128/am.27.4.678-687.1974 PMC380117

[45] Yassin H, Velthuis AGJ, Boerjan M, van Riel J (2009) Field study on broilers' first-week mortality. Poultry Science 88: 798–804. 10.3382/ps.2008-00292 19276423

[46] Le Gall T, Clermont O, Gouriou S, Picard B, Nassif X, et al (2007) Extraintestinal virulence is a coincidental by-product of commensalism in B2 phylogenetic group Escherichia coli strains. Molecular Biology and Evolution 24: 2373–2384. 17709333 10.1093/molbev/msm172

[47] Nowrouzian FL, Wold AE, Adlerberth I (2005) Escherichia coli strains belonging to phylogenetic group B2 have superior capacity to persist in the intestinal microflora of infants. Journal of Infectious Diseases 191: 1078–1083. 15747243 10.1086/427996

[48] Nowrouzian FL, Adlerberth I, Wold AE (2006) Enhanced persistence in the colonic microbiota of Escherichia coli strains belonging to phylogenetic group B2: role of virulence factors and adherence to colonic cells. Microbes and Infection 8: 834–840. 16483819 10.1016/j.micinf.2005.10.011

[49] Crhanova M, Hradecka H, Faldynova M, Matulova M, Havlickova H, et al (2011) Immune Response of Chicken Gut to Natural Colonization by Gut Microflora and to Salmonella enterica Serovar Enteritidis Infection. Infection and Immunity 79: 2755–2763. 21555397 10.1128/IAI.01375-10 PMC3191970

[50] Sabri M, Leveille S, Dozois CM (2006) A SitABCD homologue from an avian pathogenic Escherichia coli strain mediates transport of iron and manganese and resistance to hydrogen peroxide. Microbiology-Sgm 152: 745–758. 10.1099/mic.0.28682-0 16514154

[51] van Elsas JD, Semenov AV, Costa R, Trevors JT (2011) Survival of Escherichia coli in the environment: fundamental and public health aspects. Isme Journal 5: 173–183. 20574458 10.1038/ismej.2010.80 PMC3105702

[52] Cortes MAM, Gibon J, Chanteloup NK, Moulin-Schouleur M, Gilot P, et al (2008) Inactivation of ibeA and ibeT results in decreased expression of type 1 fimbriae in extraintestinal pathogenic Escherichia coli strain BEN2908. Infection and Immunity 76: 4129–4136. 18591231 10.1128/IAI.00334-08 PMC2519445

[53] Huang SH, Wass C, Fu Q, Prasadarao NV, Stins M, et al (1995) Escherichia-coli invasion of brain microvascular endothelial-cells in-vitro and in-vivo - molecular-cloning and characterization of invasion gene ibe10. Infection and Immunity 63: 4470–4475. 7591087 10.1128/iai.63.11.4470-4475.1995 PMC173636

[54] Johnson TJ, Wannemuehler Y, Doetkott C, Johnson SJ, Rosenberger SC, et al (2008) Identification of Minimal Predictors of Avian Pathogenic Escherichia coli Virulence for Use as a Rapid Diagnostic Tool. Journal of Clinical Microbiology 46: 3987–3996. 18842938 10.1128/JCM.00816-08 PMC2593276

[55] Schouler C, Schaeffer B, Bree A, Mora A, Dahbi G, et al (2012) Diagnostic Strategy for Identifying Avian Pathogenic Escherichia coli Based on Four Patterns of Virulence Genes. Journal of Clinical Microbiology 50: 1673–1678. 22378905 10.1128/JCM.05057-11 PMC3347144

[56] Ewers C, Li GW, Wilking H, Kiessling S, Alt K, et al (2007) Avian pathogenic, uropathogenic, and newborn meningitis-causing Escherichia coli: How closely related are they? International Journal of Medical Microbiology 297: 163–176. 17374506 10.1016/j.ijmm.2007.01.003

[57] Rodriguez-Siek KE, Giddings CW, Doetkott C, Johnson TJ, Fakhr MK, et al (2005) Comparison of Escherichia coli isolates implicated in human urinary tract infection and avian colibacillosis. Microbiology-Sgm 151: 2097–2110. 10.1099/mic.0.27499-0 15942016

[58] Wells LL, Lowry VK, DeLoach JR, Kogut MH (1998) Age-dependent phagocytosis and bactericidal activities of the chicken heterophil. Developmental and Comparative Immunology 22: 103–109. 9617587 10.1016/s0145-305x(97)00024-4

[59] Withanage GSK, Kaiser P, Wigley P, Powers C, Mastroeni P, et al (2004) Rapid expression of chemokines and proinflammatory cytokines in newly hatched chickens infected with Salmonella enterica serovar typhimurium. Infection and Immunity 72: 2152–2159. 15039338 10.1128/IAI.72.4.2152-2159.2004 PMC375210

[60] Withanage GSK, Wigley P, Kaiser P, Mastroeni P, Brooks H, et al (2005) Cytokine and chemokine responses associated with clearance of a primary Salmonella enterica serovar Typhimurium infection in the chicken and in protective immunity to rechallenge. Infection and Immunity 73: 5173–5182. 16041035 10.1128/IAI.73.8.5173-5182.2005 PMC1201213

[61] Casadevall A, Pirofski LA (2001) Host-pathogen interactions: The attributes of virulence. Journal of Infectious Diseases 184: 337–344. 11443560 10.1086/322044

